# Integration and Validation of a Natural Language Processing Machine Learning Suicide Risk Prediction Model Based on Open-Ended Interview Language in the Emergency Department

**DOI:** 10.3389/fdgth.2022.818705

**Published:** 2022-02-02

**Authors:** Joshua Cohen, Jennifer Wright-Berryman, Lesley Rohlfs, Douglas Trocinski, LaMonica Daniel, Thomas W. Klatt

**Affiliations:** ^1^Clarigent Health, Mason, OH, United States; ^2^Department of Social Work, College of Allied Health Sciences, University of Cincinnati, Cincinnati, OH, United States; ^3^WPP Emergency Services, Raleigh, NC, United States; ^4^WPP Clinical Research, Raleigh, NC, United States; ^5^Behavioral Health Network, Raleigh, NC, United States

**Keywords:** suicide, machine learning, natural language processing, emergency department (ED), risk assessment, mental health, validation, feasibility & acceptability

## Abstract

**Background:**

Emergency departments (ED) are an important intercept point for identifying suicide risk and connecting patients to care, however, more innovative, person-centered screening tools are needed. Natural language processing (NLP) -based machine learning (ML) techniques have shown promise to assess suicide risk, although whether NLP models perform well in differing geographic regions, at different time periods, or after large-scale events such as the COVID-19 pandemic is unknown.

**Objective:**

To evaluate the performance of an NLP/ML suicide risk prediction model on newly collected language from the Southeastern United States using models previously tested on language collected in the Midwestern US.

**Method:**

37 Suicidal and 33 non-suicidal patients from two EDs were interviewed to test a previously developed suicide risk prediction NLP/ML model. Model performance was evaluated with the area under the receiver operating characteristic curve (AUC) and Brier scores.

**Results:**

NLP/ML models performed with an AUC of 0.81 (95% CI: 0.71–0.91) and Brier score of 0.23.

**Conclusion:**

The language-based suicide risk model performed with good discrimination when identifying the language of suicidal patients from a different part of the US and at a later time period than when the model was originally developed and trained.

## Introduction

Suicide remains the 10^th^ leading cause of death in the United States overall (1). Although recent data suggest that suicide rates from 2018 to 2019 decreased 2.1%, 12 million adults reported suicidal thoughts, 3.5 million reported a suicide plan, and 1.4 million reported a suicide attempt (1). A report of the Joint Commission of the Accreditation of Healthcare Organizations (2) revealed that suicide screening in emergency departments (ED) may be an important intercept point for identifying those at risk and connecting them to care, however, current screening tools may not provide person-centered risk identification with consistent sensitivity (3). Therefore, innovative and person-centered methods of identifying suicide risk are a critical need.

Despite decades of research, suicide rates have steadily risen, while detection of who is at highest risk for death has not improved (4, 5). A recent meta-analysis suggests that current methods of predicting risk for suicide death are no better than 50% or random chance (6, 7). Although theories exist about why people die by suicide (8–11) they have not aided the development of adequate predictive models for reducing death rates, nor have they resulted in screening instruments that have sufficient predictive value (7, 12).

Natural language processing (NLP) and machine learning (ML) have shown promise in identifying suicide risk (13). However, many of these NLP methods have been applied to analyzing social media texts or in non-clinical settings (14, 15). Additionally, NLP methods that have been used with clinically significant language data have been gathered from existing records (16, 17) instead of using interviews at point-of-contact. A recent meta-analysis suggests that NLP holds promise for accurate detection of suicide risk when deployed within the assessment process (18), as most studies showed an AUC >0.90. However, the authors assert more work should be done using real-time language data collection for translation into clinical practice. A study by Chakravarthula et al. used NLP with military couples to identify suicide risk by analyzing couples' conversations (19). The investigators found that the model predicted risk better than random chance in all risk categories. A 2021 study using smartphones to collect language data from 588 veterans also successfully determined suicide risk with 0.86 sensitivity, 0.70 specificity and an AUC >0.80 (20).

Previous research of the technology used in this study was conducted primarily in the Midwestern United States from 2013 to 2015 by Pestian et al., and used language collected through interviews of suicidal and non-suicidal adults and adolescents to “train” an NLP/ML model to identify language features of suicidal individuals (21, 22). In these studies, called the Adolescent Controlled Trial (ACT) and Suicide Thought Markers (STM) study, a total of 160 suicidal participants, 126 non-suicidal participants with mental illness, and 153 non-suicidal participants without mental illness were enrolled across three hospitals' EDs and psychiatric units. Support vector machine (SVM) models were trained to identify suicidal participants (case) vs. non-suicidal participants with and without mental illness (control) with an area under the receiver operating characteristic curve (AUC) of 0.69–0.93 depending on the cross-validation approach used (21, 22). While these models have performed well-across a series of studies, many factors could influence language potentially affecting model generalizability and validation when applied at later time periods or in separate geographic regions.

Validation is one of the most important steps during model development and provides evidence a model will perform as expected with a new dataset and in new settings. Broadly, internal validation tests a model with the development data through different protocols such as cross-validation, where data are segmented into groups or “folds” and a model is iteratively trained on all but one fold, with performance examined on the hold-out. Internal validation procedures may produce accurate models and demonstrate a proof of concept, however, if a model is to be used in more critical settings such as medical or psychiatric settings, additional testing should be done. External validation tests a model with data collected separate from the data used to develop the model, where one or more variables (e.g., time point or location) are changed to ensure model performance remains acceptable (23, 24). Additionally, clincal NLP models pose unique challenges to algorithm portability – the ability to apply a model in diverse settings – due to the need to assemble clinical corpora, site-specific reporting structures, and the idiosyncrasies of language use (25, 26), underscoring the need for external validation.

The purpose of this pilot study was to (1) determine if the interview process to collect language for an NLP/ML model could be integrated into two EDs in the Southeastern United States, and (2) evaluate model performance on language from persons in a different geographic region than where the original model was developed. Notably, this study was conducted during the COVID-19 pandemic, which has led to increased stress and isolation and has likely impacted language use (27, 28). External validation is especially important during COVID-19 for a spoken language-based model because the data used to develop our model was collected prior to the pandemic, meaning the model cannot recognize the pandemic as a factor impacting suicidal risk. We do not know when the pandemic will end and therefore, must understand any limitations of these methods in this setting.

## Methods

The protocol was approved by the health system's institutional review board which oversaw the study conducted at the two EDs. All subjects gave informed consent in accordance with the Declaration of Helsinki before they participated in the study.

### Study Staff and Participants

#### Study Staff

The study staff was composed of four Behavioral Health Licensed Clinicians (BHLC) for case participants and two clinical research coordinators (CRC) for control participants. Study staff completed online training to learn study procedures, principles of human subject protection, and good clinical practice.

#### Participants and Case-Control Definitions

Criteria for participant recruitment were: (1) a patient receiving ED services, (2) age 18–65, (3) able to provide informed consent, and (4) English as a primary language. Case participants presented to an ED with suicidal ideation or a suicide attempt within the last 30 h. Control participants presented to an ED for any non-suicide related condition and had no lifetime history of suicide risk or mental health diagnosis.

### Study Design

This case-control study sought to externally validate an NLP/ML model trained on data collected from the ACT and STM studies, and therefore aimed to keep procedures as consistent as possible between those studies. Because there is no generally accepted approach to estimate sample sizes for development and validation of risk prediction models (23), sample size was determined from previous studies and feasibility considerations. Previous studies enrolled 30–44 case and control participants per site, which allowed performance estimates with acceptable precision (21, 22).

**Figure 1:**
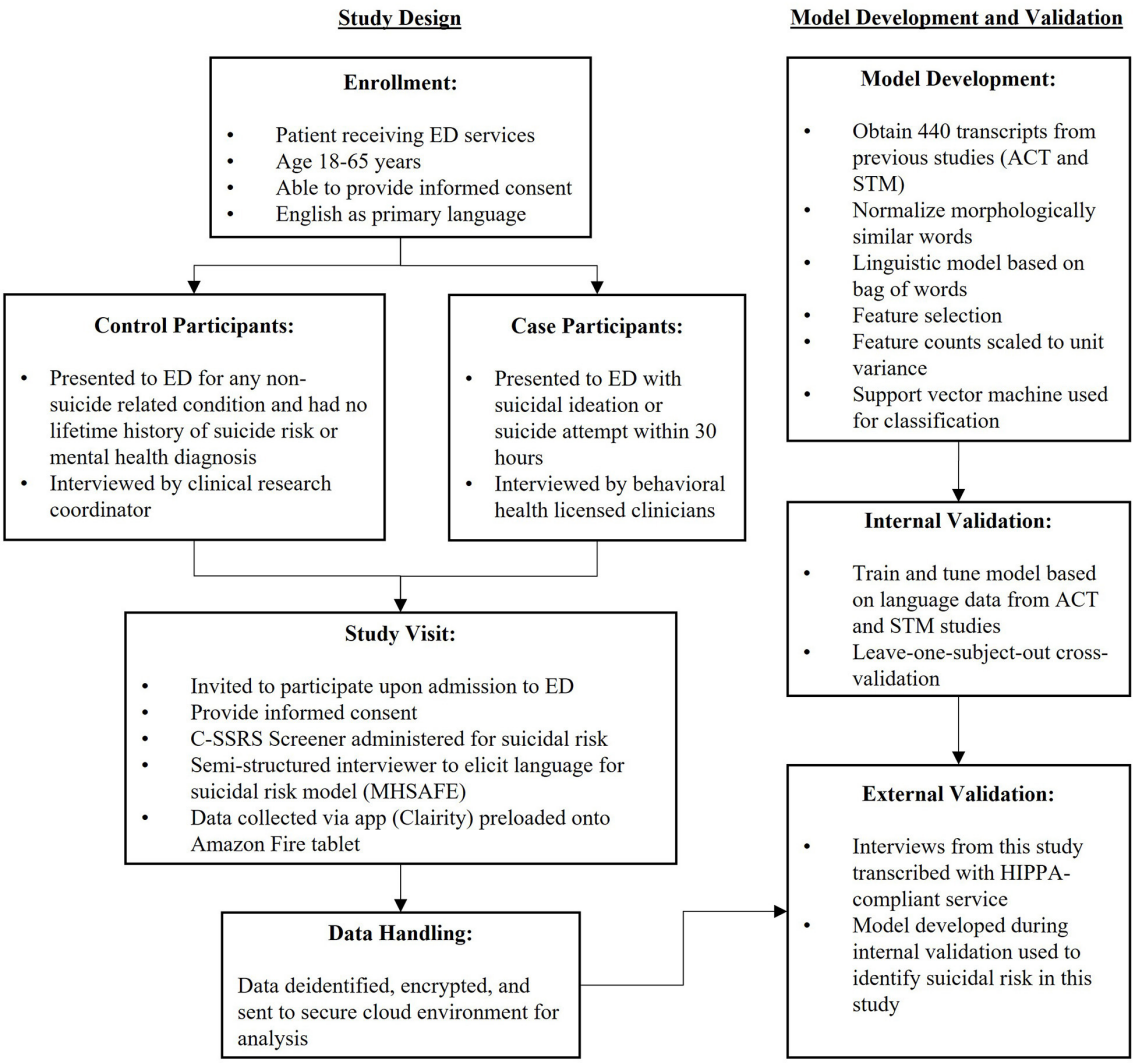
Schematic of study and modeling procedures.

Upon admission to the ED, patients were invited to participate in the study and the C-SSRS Screener was administered (see Figure 1). The C-SSRS Screener is a structured interview based on the full-length version (29), and is designed to measure suicidal ideation and suicidal behaviors on an ordinal scale. In a multi-site emergency department study, the C-SSRS demonstrated high sensitivity and specificity for classifying suicidal behavior, as well as strong internal consistency and predictive validity (11). The C-SSRS Screener is composed of two initial questions and four follow up questions if the participant answers “yes” to question 2. The first five questions relate to the severity of suicidal ideation and ask about the past month. These questions include: (1) “Have you wished you were dead or wished you could go to sleep and not wake up?” (2) “Have you actually had any thoughts of killing yourself?” (3) “Have you been thinking about how you might do this?” (4) “Have you had these thoughts and had some intention of acting on them?” and (5) “Have you started to work out or worked out the details of how to kill yourself? Do you intend to carry out this plan?” The final question measures the presence of suicidal behavior over the lifetime and the past 3 months: (6) “Have you ever done anything, started to do anything, or prepared to do anything to end your life?”

The study staff then conducted a semi-structured interview comprised of pre-established probes (renamed MHSAFE - hope, secrets, anger, fear, and emotional pain - from the “Ubiquitous Questionnaire”), and used an application installed on a tablet to record the interview. The original Ubiquitous Questionnaire was developed with experts in suicide research in order to elicit emotional language related to salient variables of hope, secrets, anger, fear, and emotional pain (30). Study staff were instructed to ask questions about participants' current feelings on all five areas, for example, “tell me about your hope?” or “are there secrets in your life?” The interviews in previous research lasted 8.1 ± 4.5 min (21). A HIPAA- compliant service was used to manually transcribe the recorded interviews. Scores from the suicide risk model were not returned to clinicians in this study because they were generated asynchronously from manual transcripts after care determinations had been made, and because the purpose of this study was to evaluate model validity.

### Data Analysis

All analysis was performed using the Python programming language [version 3.7.5; (31)]. The open-source Python libraries Pandas [version 1.1.2; (32, 33)], Numpy [version 1.18.5; (34, 35)], scikit-learn [version 0.23.2; (36)], Matplotlib [version 3.7.5; (37)], SciPy [version 1.5.2; (38)], and NLTK [version 3.2.2; (39)] were used for data analysis and all NLP/ML model building. Student's *t*-tests were performed with SciPy's ttest_ind function. The study adhered to the TRIPOD (Transparent Reporting of a multivariable prediction model for Individual Prognosis or Diagnosis) statement for reporting (23). Figure 1 shows model development and validation procedures.

The NLP/ML pipeline used in this study followed similar techniques used by Pestian et al., focused on the term frequency of n-grams (contiguous sequence of n number of words) and SVMs (21, 22, 40–42). The Porter Stemmer algorithm was applied to participant language to normalize morphologically related terms (43). Language was tokenized by splitting on white spaces, and 1–3 continuous sequences of words were used as features. Scikit-learn's SelectKBest function was used to identify features with the highest ANOVA F-value, with the number of features selected as a tunable hyperparameter to optimize model performance, including 1024, 2048, 4096, and “all” features. Selected features were then scaled to unit variance. During SVM tuning, hyperparameters considered include: the regularization parameter (C), the kernel (radial basis function and linear kernels), the kernel coefficient (gamma, if applicable), and the class weight. Additional details on NLP/ML methods may be found in previous work (42). We have evaluated the performance of Logistic Regression and XGBoost models in previous work and found comparable performance across models (42), and therefore decided to continue with the SVM used previously (21). A comparison of different models is beyond the scope of this paper.

During model training using ACT and STM data, the only input was the participant's language, labeled as case or control. During model testing using data from this study, a participant's language was fed into the model, and a probability for belonging to the case group was returned. Model performance was then evaluated by comparing model predictions to the participant's labeled group (case or control) and calculating the AUC and Brier score, two recommended measures to provide a more complete picture of model performance (23). AUC values range from 0.5 (random chance) to 1.0 (perfect model). The Brier score is a measure of model calibration and ranges from 0 to 1, where low scores indicate less discrepancy between predicted probabilities and outcomes. If a model is calibrated, then its output probabilities convey meaningful information. For example, a calibrated model that returns a 30% chance of having a disease means of all tests that received a score of about 30%, 30% of them had the disease (44). Thus, the AUC is a preferred metric for model evaluation because it gives an overall measure of model discrimination without imposing probability thresholds for classification that can result in a loss of information (23, 45).

Additional classification metrics were calculated for different probability thresholds to classify a new interview as positive (suicidal). Metrics considered include sensitivity, specificity, positive predictive value (PPV), and negative predictive value (NPV). Sensitivity and specificity measure how well a test identifies true positives and true negatives, respectively. PPV measures the proportion of true positives out of all who test positive, and NPV measures the proportion of true negatives out of all who test negative.

### Internal Validation

Data from the ACT and STM studies have been internally validated in separate publications and report AUCS from 0.69 to 0.93 depending on the features (acoustic or linguistic), participants included (controls, those with mental illness not suicidal, and suicidal), and cross-validation technique (leave-one-subject-out and leave-one-site-out) (21, 22). For internal validation in this study, we performed a leave-one-subject-out cross-validation for the combined ACT and STM dataset comprised of 440 participant interviews using only linguistic data. This cross-validation technique provides the most optimistic internal validation performance by iteratively training a model on all but one interview and predicting to which class the hold-out interview belongs. We focused exclusively on linguistic features because previous work compared linguistic and acoustic (e.g., fundamental frequency) features and found acoustic features did not improve predictive value for this dataset (21).

### External Validation

For external validation, a model was trained and tuned on the complete ACT and STM dataset (controls, suicidal, and mentally ill), and then used to predict suicidal risk from the language samples collected in this study. The suicidal risk for model performance was determined by the study arm to which the participant was assigned. For case participants, this suicidal risk can be characterized by recent suicide death-related ideations or behaviors that lead to their admission to the ED. Language samples were collected within 30 h of admission.

## Results

### Population and Data Collection

Between September and December 2020, 70 participants were enrolled, each providing one session recording. The control arm of the study interviewed 33 individuals and the case arm interviewed 37. Table 1 shows a summary of participant descriptive statistics. A significant difference in participant word count between case and control interviews is shown in Table 1 (*t*-test, *p* = 0.02), although this difference is not present in our training data (*p* = 0.77).

**Table 1:** Participant descriptive statistics.

**Variable**	**Control**	**Case**	**Both**
Enrolled	33	37	70
Average age (SD)	41.2 (12.5)	41.1 (12.8)	40.1 (12.5)
**Gender**			
Male (%)	18 (54.50%)	20 (54.10%)	38 (54.30%)
Female (%)	15 (45.50%)	16 (43.20%)	31 (44.30%)
Transgender (%)	0 (0%)	1 (2.70%)	1 (1.40%)
**Race**			
White or Caucasian (%)	15 (45.50%)	20 (54.10%)	35 (50.00%)
Black or African American (%)	15 (45.50%)	17 (45.90%)	32 (45.70%)
Other (%)	3 (9.10%)	0 (0%)	3 (4.30%)
Average interview length (min) (SD)	7.8 (3.1)	7.1 (3.1)	7.4 (3.1)
Average participant word count (SD)	723 (401)	485 (432)	593 (431)

All control participants responded negatively to all questions on the C-SSRS Screener and confirmed no history of suicidality or a mental illness diagnosis. Table 2 describes how participants in the case group answered the C-SSRS Screener. One case participant answered negatively to all questions on the C-SSRS Screener. This participant was still considered a case in this study because they were admitted to the ED for suicidal ideation with a diagnosis for suicidal ideation.

**Table 2:** Summary of case participant answers to the C-SSRS screener.

**C-SSRS question**	**Question topic**	**N**	**%**
**Past month**			
-	No suicidal ideation (SI)	1	3%
1	Wish to be dead	35	95%
2	Non-specific active SI	32	86%
3	SI with methods	26	70%
4	Suicidal intent	24	65%
5	Suicidal intent with plan	24	65%
**Lifetime**			
6a	Suicidal behavior	26	70%
**Past 3 months**			
6b	Suicidal behavior	15	41%

### Internal Validation

The NLP/ML model reached an AUC of 0.82 (95% CI = 0.78–0.86) and a Brier score of 0.16 using a leave-one-subject-out cross-validation technique for the 440 subject interviews in the ACT and STM training dataset. This model performed optimally with 4096 n-gram features and a linear kernel. Table 3 shows classification performance metrics for different probability risk thresholds for a positive (suicidal) prediction. The top ten model features for case and control predictions are available in Supplementary Table 1.

**Table 3:** Internal and external validation classification performance at different risk thresholds.

**Risk threshold^a^**	**Sensitivity^b^ (95% CI)**	**Specificity^c^ (95% CI)**	**PPV^d^ (95% CI)**	**NPV^e^ (95% CI)**
**Internal validation**
≥10%	0.93 (0.88–0.96)	0.39 (0.33–0.45)	0.47 (0.41–0.52)	0.91 (0.84–0.95)
≥20%	0.85 (0.79–0.90)	0.57 (0.51–0.62)	0.53 (0.47–0.59)	0.87 (0.81–0.91)
≥35%	0.73 (0.66–0.80)	0.72 (0.67–0.77)	0.60 (0.53–0.67)	0.82 (077–0.87)
≥50%	0.63 (0.55–0.70)	0.84 (0.79–0.87)	0.69 (0.61–0.76)	0.80 (0.75–0.84)
**External validation**
≥10%	0.81 (0.66–0.91)	0.55 (0.38–0.70)	0.67 (0.52–0.79)	0.72 (0.52–0.86)
≥20%	0.73 (0.57–0.85)	0.76 (0.59–0.87)	0.77 (0.61–0.88)	0.71 (0.55–0.84)
≥35%	0.65 (0.49–0.78)	0.88 (0.73–0.95)	0.86 (0.69–0.94)	0.69 (0.54–0.81)
≥50%	0.54 (0.38–0.69)	0.94 (0.80–0.98)	0.91 (0.72–0.97)	0.65 (0.50–0.77)

a *Model scores equal to or above this value are classified as suicidal*.

b *Sensitivity = true positives divided by sum of true positives and false negatives*.

c *Specificity = true negatives divided by sum of true negatives and false positives*.

d *Positive predictive value = true positives divided by sum of true positives and false positives*.

e *Negative predictive value = true negatives divided by sum of true negatives and false negatives*.

### External Validation

The NLP/ML model trained and tuned on the ACT and STM dataset reached an AUC of 0.81 (95% CI = 0.71–0.91) and a Brier score of 0.23 when predicting suicidal risk on the 70 patient interviews collected in this study. Table 3 shows classification performance metrics for different probability risk thresholds for a positive (suicidal) prediction. Receiver operating characteristic curves for internal and external validation are available as Supplementary Figure 1.

## Discussion

In this study, we found it feasible to integrate technology and procedures to collect language for a suicide risk prediction model into the ED workflow. Additionally, a follow-up study using thematic analysis of BHLC semi-structured interviews (46) revealed that minor issues such as logging into the system were easily overcome, and the use of the app to consent the participant and record the session were well-managed in the workflow. The clinicians also reported that in comparison to standardized scales (screening as usual), use of the probes did not impede the ED process, gleaned more information about the person's mental state, and the probes were reported as a more person-centered approach to screening for suicide risk. Although a five-to-ten-minute interview may take longer than a self-report brief scale, more usable data for clinical decision-making were reportedly obtained via the probes (46).

The model, trained on language from the ACT and STM studies, performed with good discrimination when identifying the language of suicidal vs. non-suicidal participants in this study. As a variable, language is influenced by time period, location, and large-scale events, such as the COVID-19 pandemic (28). Therefore, we were uncertain how well a language-based model trained on data collected in the Midwest from 2013 to 2015 would perform on the language collected in this study. Interestingly, despite these factors, model performance was similar to previous studies [AUC range 0.69–0.93; (21, 22)], supporting geographic and temporal validation.

As a performance metric, AUC may be interpreted as the probability randomly selected case (suicidal) participants will receive a higher probability score from the model than randomly selected control (non-suicidal) participants (47). The classification metrics in Table 3 require defining a threshold for classification, although as mentioned, setting a risk threshold for classification may result in the loss of clinically relevant information if a model is calibrated. Brier scores were used to measure calibration in this study, and we found the Brier score increased 0.07 during external validation, indicating the model may be less calibrated in this setting. Indeed, during internal validation, sensitivity and specificity are roughly equal at a risk threshold of 35% but are equal at a lower risk threshold of 21% for external validation, indicating interviews in this study receive a lower probability of being suicidal, overall. Thus, while model performance as measured by the AUC is similar to the internal validation, it may be that models require additional calibration when applied in new settings.

While a change in geography likely impacts the need for additional calibration, we expect temporal effects play a larger role on the model's output. Data drift- the degradation of model performance over time, is a common concern when developing models, and is due in part to a change in relation between features (e.g., language) and the outcome of interest (e.g., suicidal risk). Broadly, the language features in the model may be categorized as content words (i.e., what someone talks about) and function words (i.e., how someone speaks). While the underlying concept behind the MHAFE interview is to elicit language about universally relatable topics (30), and some content and function word usage might be time-agnostic, participants in this study discussed topics that did not exist in 2015, such as the COVID-19 pandemic. Therefore, the model was unaware of additional factors that could contribute to an individual's suicidal risk, potentially lowering the model's score.

More advanced natural language processing techniques may offer a solution to generalize over broader geographical and temporal settings. Word vectors encode words into a high dimensional space (50–300 dimensions) that retain semantic meaning and have demonstrated state-of-the-art performance on many language tasks (48–50). However, the semantic meaning encoded in word vectors is derived from specific corpora (e.g., all of Wikipedia) and in many cases has been found to also retain biases (50–52). Additionally, models using word vectors may struggle to explain the reason for a specific prediction, which is becoming required for clinical decision support systems (53, 54). For these reasons, we have focused primarily on more traditional NLP/ML techniques but will explore more advanced techniques as more tools for explainable AI and AI safety become available.

Nevertheless, as with all screening and diagnostic tools, there will always be variables that can impact results, and how a tool was developed should be considered when interpreting results. For example, there have been gender imbalances in clinical research (55, 56), so caution must be taken when generalizing results across the underrepresented gender. Similarly, clinicians using a language-based tool to identify suicide risk should be trained to understand its limitations, such as this model's inability to recognize language related to COVID-19. We have been collecting virtual interviews throughout the pandemic with participants from every United States geographical region to update our model and better understand other potential limitations related to geo-temporal effects.

Traditional theories to explain suicide follow an ideation-to-action framework, where there is a linear progression from suicidal ideation to suicidal behavior. Recent work, however, has highlighted these theories of suicide are incomplete (7, 57, 58). A meta-analysis of 71 studies examining the relation between suicide ideation and later suicide found ~60% of individuals who either engaged in suicidal behaviors or later died by suicide did not express suicidal ideation (58). These findings have led to the suggestion of multiple pathways that can lead to suicidal behavior, one of which includes a subgroup of individuals who do not first experience active suicidal ideation before suicidal behavior (57). Therefore, suicide screening tools built upon ideation-to-action theories, such as the C-SSRS Screener used in this study, likely miss a significant portion of individuals who may later die by suicide. Longitudinal studies could help us understand if patient language may help in identifying these false negatives of traditional scales.

Clinically, the results of this study suggest that the MHSAFE process for screening suicide risk may add to the limited options available to providers. In this ED study, the BHLC reported ease of integration of the technology, and that this qualitative, person-centered, interview approach to suicide risk screening has added clinical benefit, such as allowing the patient to open up and provide more information and nuance (46). Future versions of the tool will provide a return of suicide risk result that can be used in clinical decisions (further assessment, safety planning).

### Limitations and Future Directions

Although these findings align with previous studies, some limitations should be noted. First, the use of BHLCs for case interviews and CRCs for control interviews could have influenced the model's ability to classify participant interviews. All study staff received the same training on the MHSAFE interview, but individual interviewer styles, previous experience, and being unblinded to outcomes could affect interviews. This study sought to externally validate a model developed on data from the ACT and STM studies, where CRCs interviewed both case and control participants. The consistency of model performance across the internal and external validation sets suggests the interviewer does not play a significant role in the model's ability to identify the language of suicidal individuals. Notably, the present approach is more reflective of potential real-world applications, supporting ecological validity.

Additional limitations include small sample size, which limited representation of gender and race, which may impact generalizability. Lastly, the use of volunteer participants could have biased clinician experience and model performance.

Future studies will analyze how a return of model results to clinicians impacts clinical decision making and risk-treatment alignment. We are also investigating techniques to autonomously conduct the MHSAFE interview using an automated voice response system. Lastly, this model was developed on US English, and future studies will focus on the model's validity with different English dialects as well as the development of models for different languages.

## Conclusions

A brief interview can be successfully implemented into two EDs and NLP/ML models can predict suicide risk from patient language with good discrimination. A strength of this study is that it was conducted in a separate geographic region and at a later time period, supporting generalizability. Screening for suicide can be an open-ended and dynamic process, and these findings have highlighted an opportunity for identifying suicide risk using a person's language.

## Data Availability Statement

The datasets presented in this article are not readily available because the dataset contains confidential health-related data that cannot be shared. These data will be made available for research purposes only to any researcher who meet criteria for access to confidential data based on relevant Institutional Review Boards. Requests to access the datasets should be directed to research@clarigenthealth.com.

## Ethics Statement

The studies involving human participants were reviewed and approved by WakeMed Health & Hospitals' Institutional Review Board. The patients/participants provided their written informed consent to participate in this study.

## Author Contributions

JC and JW-B wrote the manuscript. JC performed statistical analysis on the corpora. DT is the principal investigator of the WakeMed Study of Suicidal Language in the ED-Pilot. LR, LD, and TK supervised the clinical research. LR provided project administration. All authors contributed to the article and approved the submitted version.

## Funding

This study was funded by Clarigent Health.

## Conflict of Interest

JC and LR are employed full-time by Clarigent Health. JW-B is a part-time consultant and is sponsored by a grant from Clarigent Health. DT, LD, and TK are employed full-time by WakeMed Health & Hospitals. The above interests do not alter our adherence to Frontiers Media's policies. Clarigent Health and WakeMed Health & Hospitals did not influence or restrict the submission of this publication.

## Publisher's Note

All claims expressed in this article are solely those of the authors and do not necessarily represent those of their affiliated organizations, or those of the publisher, the editors and the reviewers. Any product that may be evaluated in this article, or claim that may be made by its manufacturer, is not guaranteed or endorsed by the publisher.

## Supplementary Material

Supplementary Figure 1 Receiver operating characteristic curve for internal and external validation.

Supplementary Table 1 Top 10 model features by feature weight. Feature importance was determined from the coefficients of a linear SVM kernel, trained on the ACT and STM dataset. Case features influence the model toward a case prediction, while control features influence the model toward a control prediction. The features are n-grams, and morphologically similar terms have been normalized using the Porter Stemmer algorithm (e.g., “alone” to “alon”). This model has a total of 4,096 features.

## Abbreviations

ACT: Adolescent Controlled Trial (22)
AUC: area under receiver operating characteristic curve
BHLC: Behavioral Health Licensed Clinician
C-SSRS: Columbia - Suicide Severity Rating Scale
CI: Confidence Interval
CRC: Clinical Research Coordinator
ED: Emergency Department
MHSAFE: Mental Health Hopes Secrets Anger Fear and Emotional Pain
ML: Machine Learning
NLP: Natural Language Processing
STM: Suicidal Thought Markers study (21)
SVM: Support Vector Machine.
