# Supplementary material for: Integration and Validation of a Natural Language Processing Machine Learning Suicide Risk Prediction Model Based on Open-Ended Interview Language in the Emergency Department
Source: Front Digit Health. 2022 Feb 2;4:818705. doi: 10.3389/fdgth.2022.818705 (PMC8847784; doi:10.3389/fdgth.2022.818705)
Supplement: Supplementary Figure 1 — Receiver operating characteristic curve for internal and external validation. [file Data_Sheet_1.docx]

Supplemental Table 1: Top 10 model features by feature weight

| **Case Features** | **Control Features** |
| --- | --- |
| hurt so | no no no |
| be alon | no no |
| and i need | well i dont |
| me sad | go back to |
| yeah it doe | guess no |
| thing will | too much i |
| with my famili | dont realli know |
| hm | back to |
| be here | when i go |
| the stuff that | it through |

Note. Feature importance was determined from the coefficients of a linear SVM kernel, trained on the ACT and STM dataset. Case features influence the model towards a case prediction, while control features influence the model towards a control prediction. The features are n-grams, and morphologically similar terms have been normalized using the Porter Stemmer algorithm (e.g., “alone” to “alon”). This model has a total of 4096 features.

**
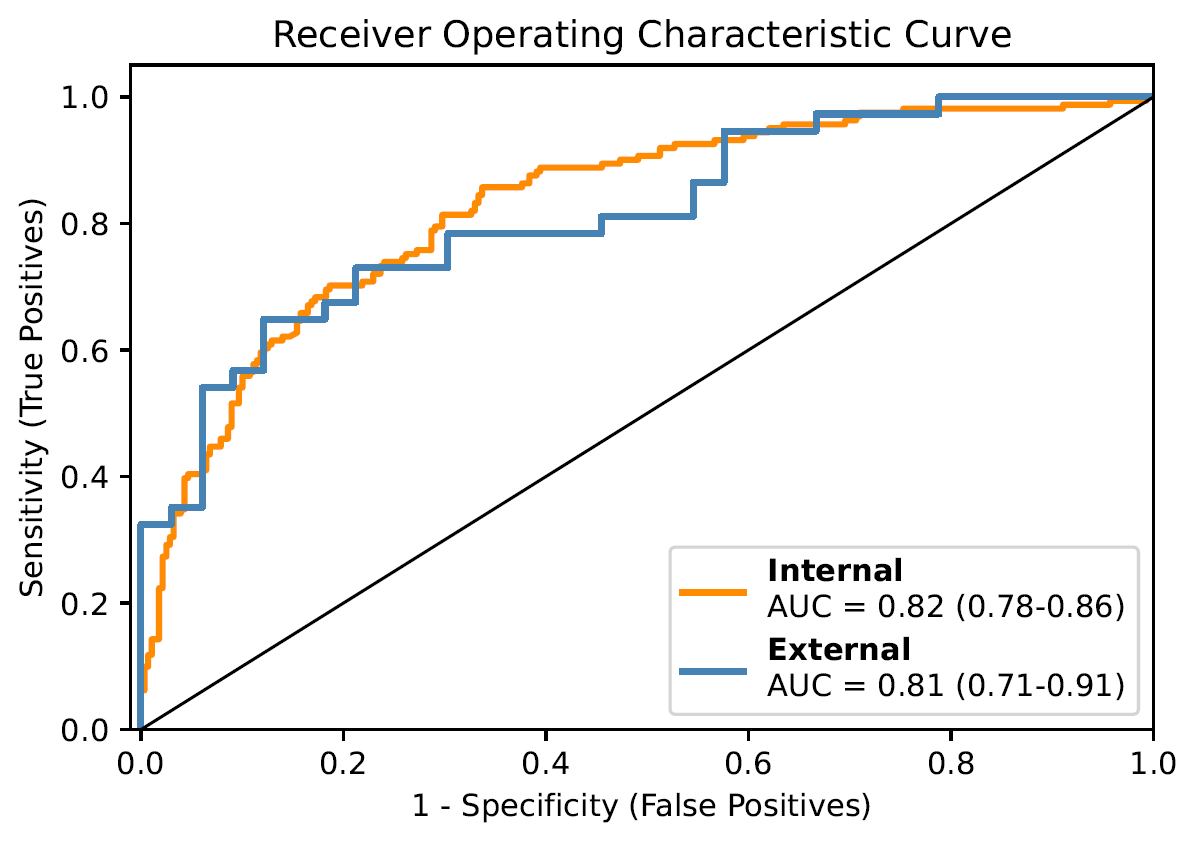
Supplemental Figure 1: Receiver operating characteristic curve for internal and external validation**
